# Machine learning models based wear performance prediction of AZ31/TiC composites

**DOI:** 10.1038/s41598-025-33417-5

**Published:** 2025-12-26

**Authors:** T. Satish Kumar, R. Raghu, Jana Petrů, S. Shalini, G. Kirubavathi, Kanak Kalita

**Affiliations:** 1 Department of Mechanical Engineering, Amrita School of Engineering, Amrita Vishwa Vidyapeetham, Coimbatore, 641112 India; 2 Department of Mechanical Engineering, Sri Ramakrishna Engineering College, Coimbatore, 641022 India; 3 Department of Machining, Assembly and Engineering Metrology, Faculty of Mechanical Engineering, VSB-Technical University of Ostrava, Ostrava, 70800 Czech Republic; 4 Department of Physics, PSG Polytechnic College, Coimbatore, 641004 India; 5 Department of Mathematics, Amrita School of Physical Sciences, Amrita Vishwa Vidyapeetham, Coimbatore, 641112 India; 6 Department of Mechanical Engineering, Rajalakshmi Institute of Technology, Chennai, 600124 India

**Keywords:** AZ31 alloy, Composite, Wear prediction, Machine learning, Engineering, Materials science

## Abstract

This study presents the fabrication of AZ31 magnesium matrix composites reinforced with 5, 10 and 15 vol% TiC particles using the Friction Stir Processing (FSP) technique and evaluates their wear behavior under varying loads (10–50 N) and sliding speeds (75–225 mm/s). The incorporation of TiC significantly enhanced the microstructural and mechanical properties of the composites. In particular, the AZ31/15 vol% TiC composite exhibited a refined grain structure with an average grain size of ~ 8 μm, compared to ~ 60 μm in the unreinforced AZ31 alloy. The same composite also demonstrated a substantial increase in hardness from 62 HV (base alloy) to 116 HV, highlighting the effectiveness of TiC reinforcement in improving strength. A key innovation of this work is the application of five machine learning (ML) algorithms, trained on experimental data using input features such as load, sliding speed and reinforcement content, to model and predict wear performance. After rigorous hyperparameter optimization, the Gradient boost algorithm achieved the highest predictive accuracy (R² = 0.9987), with errors falling within the range of experimental uncertainty. The study further includes residual analysis and computational efficiency assessment, supporting interpretable and robust AI-driven modeling. This integrated experimental-ML approach establishes a new benchmark for predictive modeling and data-driven material design in magnesium-based metal matrix composites.

## Introduction

Magnesium (Mg) alloys, owing to their low density, high specific strength and excellent machinability, have garnered significant interest in aerospace and automotive industries as potential alternatives for reducing vehicle weight and improving fuel efficiency^1^. However, the practical application of Mg alloys is constrained by inherent drawbacks such as poor wear resistance, low elastic modulus and high corrosion susceptibility^2^. To address these limitations, the incorporation of reinforcing particulates into the Mg matrix has emerged as a promising strategy, leading to the development of metal matrix composites (MMCs) with enhanced mechanical and tribological properties^3^.

Various reinforcements have been investigated in literature to improve the performance of Mg-based composites. Ceramic particles such as ZrB₂^4^, TiB₂^5^, TiC^6^, SiC^7^, B₄C^8^, TiO₂^9^, Al₂O₃^10^, BN^11^, TiN^12^, as well as carbon-based nanostructures like carbon nanotubes (CNTs)^13^ and graphene nanoplatelets (GNPs)^14^ have demonstrated significant improvements in hardness, strength and wear resistance. Additionally, low-cost industrial by-products and bio-based fillers such as fly ash^15^ and eggshell^16^ have been explored as sustainable alternatives. Among the numerous ceramic reinforcements available, TiC stands out as a particularly effective additive for improving the wear resistance of magnesium alloys. TiC possesses high hardness (~ 3200 HV), excellent thermal stability and good chemical compatibility with Mg matrices, which facilitates strong interfacial bonding and load transfer during mechanical deformation^17^. Compared to more widely studied reinforcements such as SiC and Al₂O₃, TiC exhibits superior wear resistance due to its higher intrinsic hardness and lower chemical reactivity with Mg at processing temperatures. While SiC is known for its cost-effectiveness, it tends to form interfacial reaction products like Mg₂Si, which can degrade composite performance. Similarly, Al₂O₃, although chemically stable, often suffers from weak interfacial bonding and agglomeration issues in Mg matrices. In contrast, TiC disperses more uniformly and effectively inhibits grain growth during solid-state processing like FSP, leading to refined microstructures and enhanced mechanical response. Studies have reported that Mg-TiC composites exhibit significantly lower wear rates and higher hardness than Mg-SiC and Mg-Al₂O₃ systems under identical testing conditions^18,19^. These properties make TiC an ideal candidate for high-load, high-friction applications where wear control is critical. Despite the broader interest in Mg-TiC systems, literature focused specifically on AZ31/TiC composites remains limited, highlighting the need for deeper exploration in this area. Friction Stir Processing (FSP) was selected for fabricating AZ31/TiC composites due to its ability to produce refined, defect-free microstructures with uniform TiC dispersion. As a solid-state technique, FSP avoids common issues in melt-based methods such as porosity, particle agglomeration and interfacial reactions. It ensures better interfacial integrity between TiC and the AZ31 matrix, minimizing intermetallic formation and enhancing load transfer. The severe plastic deformation during FSP also promotes dynamic recrystallization, resulting in grain refinement and improved strength and wear resistance. Compared to powder metallurgy and laser-based techniques, FSP offers better scalability, lower porosity and greater environmental compatibility.

Mg-CeO₂ composites (0.5 to 1.5 vol%) fabricated by powder metallurgy were studied by Kujur et al.^20^ for its tensile characteristics, ignition resistance and damping capacity. Their results showed that the microhardness, ignition resistance and damping capacity of pure Mg were improved by the addition of CeO₂. Kujur et al.^21^, likewise, investigated the mechanical and corrosion characteristics of pure Mg and Mg reinforced with 1 vol% CeO₂ and magnesium-zinc alloys containing 0.5% zinc and 1% 1CeO₂. Their findings showed that the Mg/1CeO₂ composite had the best corrosion resistance and better compressive strength then AZ31 alloy.

Recently there has been tremendous focus to incorporate the potential of machine learning (ML) in developing better materials. It has two major advantages:

1) the ability to predict the resultant characteristics of materials with high accuracy, including hardness^22^, tensile strength^23^ and wear rate^24^.

2) The opportunity to enhance material properties and performance, while elucidating the complex relationships among input variables, thereby allowing more informed decision-making in material design and optimization.

Though they usually need big data, ML techniques can produce encouraging outcomes even when used on small datasets. High accuracy in predicting output parameters has been shown by traditional ML models such as Decision Tree (DT), Random Forest (RF) and Support Vector Machines (SVM)^25–27^. Given its demonstrated success in predicting the results of costly and time-consuming experimental trials, ML is essential for precisely forecasting the wear performance of magnesium matrix composites. The research, however based on the current literature, shows no prediction of wear characteristics for AZ31/TiC reinforced composites.

In this work, the wear behavior of AZ31/TiC composites was forecasted using five ML models—Linear Regression (LR), Decision Tree (DT), Random Forest (RF), Gradient Boosting (GB) and Extreme Gradient Boosting (XGB). This work intends to assess their predictive capacity. In this study, AZ31/TiC composites containing 5, 10 and 15 vol% TiC were fabricated using Friction Stir Processing (FSP). The wear rates of the AZ31 alloy and its composites were evaluated under applied loads of 10, 20, 30, 40 and 50 N at sliding speeds of 75, 150 and 225 mm/s. Comprehensive analysis of the worn surfaces, wear debris and counter-face materials were carried out to identify the predominant wear mechanisms under various testing conditions. These results not only advance the understanding of wear behavior in particle-reinforced Mg alloys but also pave the way for data-driven design and deployment of lightweight, wear-resistant components in sectors such as automotive, aerospace and biomedical implants, where material efficiency and predictive performance are critical.

## Experimental study

The base material was AZ31 magnesium alloy plates with dimensions 100 × 100 × 6 mm procured from Hindalco Industries Ltd., India in hot-extruded condition (O-temper).Table 1 shows the chemical composition of AZ31 alloy. The average grain size of the unprocessed AZ31 alloy was observed to be 60 μm. The reinforcement used in this study was TiC particles with an average size of 6 to 10 μm. The titanium carbide (TiC) reinforcement particles were procured from Sigma Aldrich with a stated purity of ≥ 99.5%. The particles were used in their as-received condition without further chemical treatment. Prior to incorporation into the AZ31 alloy matrix, the TiC powder was subjected to ultrasonic dispersion in ethanol for 15 min to break down soft agglomerates and ensure better homogeneity during filling. The base metal was grooved and the required amount (5, 10 and 15 vol%, TiC) particles were filled into the groove. The selection of TiC volume fractions 5%, 10% and 15% was strategically made based on a balance between mechanical enhancement and processability in the AZ31 magnesium matrix. Literature indicates that ceramic reinforcements beyond 15–20 vol% often lead to particle agglomeration, poor ductility and tool wear during processing, especially in solid-state routes like FSP.

A pinless FSP tool was then used to set the particles inside the grooves. Friction Stir Processing (FSP) single-pass approach was performed using a cylindrically tapered WC tool, featuring a 16 mm shoulder diameter, a 5 mm pin length and a 4 mm pin diameter. To ensure efficient material mixing and consolidation, the processing parameters were maintained at a rotational speed of 1000 rpm^28,29^ a traverse speed of 20 mm/min and an applied force of 10 kN.

**Table 1:** Chemical composition of the AZ31 alloy.

Element (wt%)	Al (%)	Zn (%)	Mn (%)	Si (%)	Fe (%)	Cu (%)	Mg (balance)
Content	2.8–3.3	0.7–1.3	0.2–1.0	≤ 0.10	≤ 0.005	≤0.05	Remainder

After friction stir processing (FSP), microstructural characterization was performed on both the AZ31 alloy and the fabricated composite specimens. Transverse cross-sections, taken perpendicular to the FSP direction, were prepared using standard metallographic procedures. Initial grinding was conducted using silicon carbide (SiC) abrasive papers with progressively finer grits (220, 400, 600, 800, 1000 and 1200). This was followed by final polishing with a 0.05 μm alumina suspension on a cloth wheel to obtain a mirror-like surface finish. Subsequently, the polished samples were etched using a reagent consisting of 4 mL picric acid, 10 mL acetic acid and 70 mL ethanol to reveal microstructural features. Microstructural analyses were conducted using Scanning Electron Microscopy coupled with Energy Dispersive Spectroscope (SEM/EDS) and Optical Microscopy (Axio Scope A1, Carl Zeiss). Phase identification of the AZ31/TiC composites was conducted using X-ray diffraction (XRD) analysis performed on a Bruker D8 Advance diffractometer equipped with a Cu Kα radiation source (λ = 1.5406 Å), operated at 40 kV and 30 mA. Scans were conducted over a 2θ range of 10° to 90**°** using a step size of 0.02**°** and a counting time of 1.0 s per step. The scan rate was maintained at 2°/min and samples were rotated during scanning to improve signal uniformity.

Hardness measurements were conducted in accordance with ASTM E92 standard procedures using a Vickers microhardness tester (Mitutoyo HM-200) with an applied load of 1 kg. For each specimen, seven indentations were taken at various locations and the average of these readings was reported as the final hardness value. The wear behavior of the AZ31/TiC composites under dry sliding conditions was evaluated using a pin-on-disc tribometer (TR-20LE, Ducom Instruments)in accordance with ASTM G99 – Standard Test Method. Prior to wear testing, all pin specimens and counterface discs were ultrasonically cleaned in acetone for 10 min to remove residual oils, debris and contaminants. After cleaning, the samples were dried using compressed air and handled using powder-free gloves to prevent surface contamination. The test employed a cylindrical pin made from the fabricated AZ31/TiC composite samples, with dimensions of 10 mm diameter and 30 mm height. The counterface material was a hardened AISI 420 stainless steel disc with a hardness of ~ 55 HRC. The counterface surface was ground to achieve an average surface roughness (Ra) of 0.15 μm, measured using a contact profilometer (Mitutoyo SJ-410). Testing was carried out under ambient laboratory conditions with a room temperature of approximately 33 °C and relative humidity of ~ 65%. The counterface roughness was verified prior to each run to ensure consistent initial conditions. The tests were conducted over a fixed sliding distance of 1500 m, with varying normal loads (10–50 N) and sliding speeds (75–225 mm/s). The volume loss (mm³) was calculated using the formula given below. To understand the wear mechanisms, worn surfaces were examined using SEM. Volume loss calculation for Pin-on-Disc Test (Cylindrical flat-ended pin) is as given below.

$$\:V\:=\:\varDelta\:m\:/\:\rho\:$$

where,$\:\varDelta\:m$is Mass loss (mg) and$\:\rho\:$is Density of the material (mg/mm³).

**Fig. 1:**
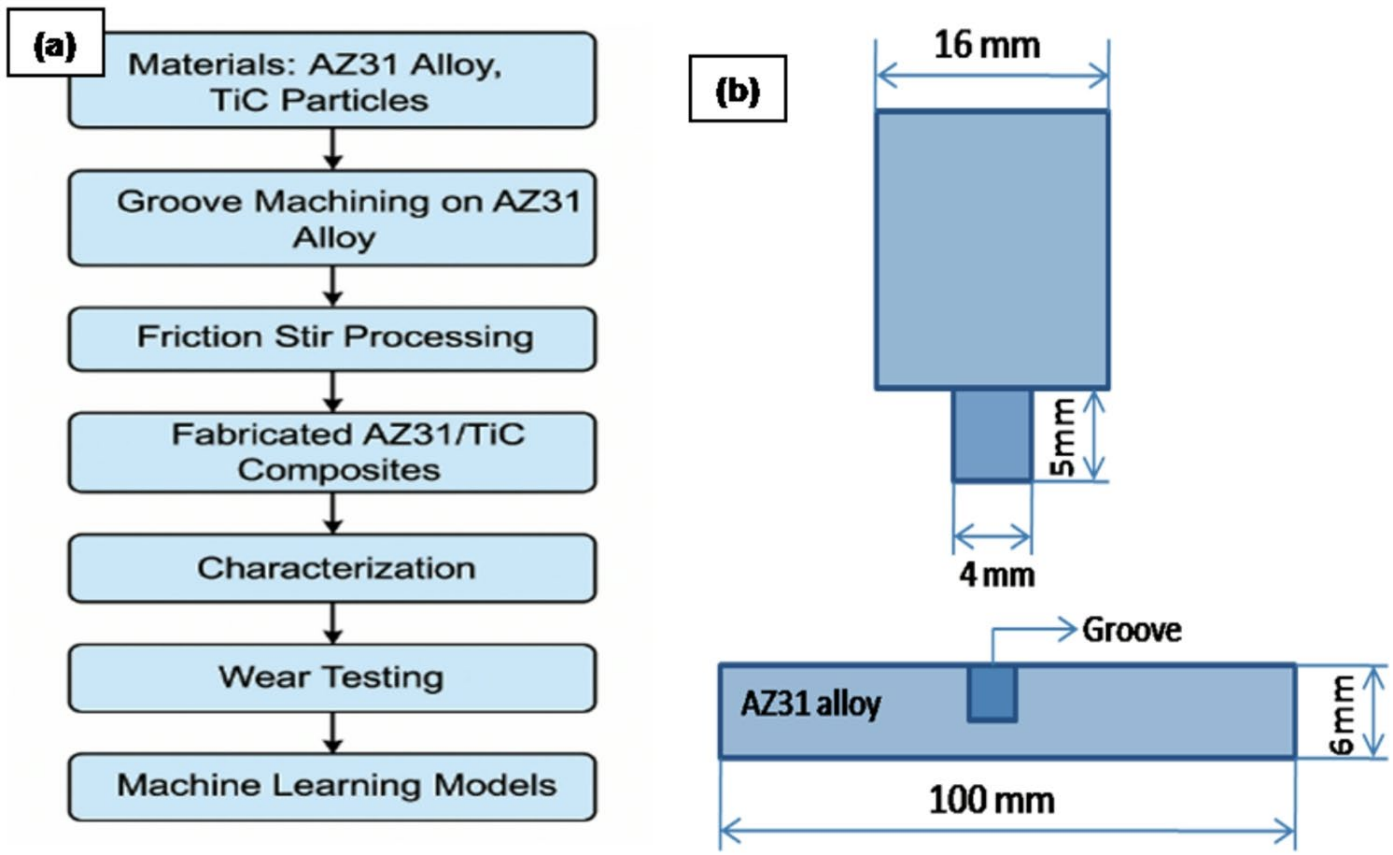
(**a**) Schematic flowchart illustrating the fabrication and characterization process and (**b**) Geometry of the FSP tool.

The flowchart (Fig. 1a &b) outlines a systematic approach to synthesizing and evaluating TiC-reinforced AZ31 composites, emphasizing process parameters and property assessments.

## Results and discussion

### Microstructure characterization

**Fig. 2:**
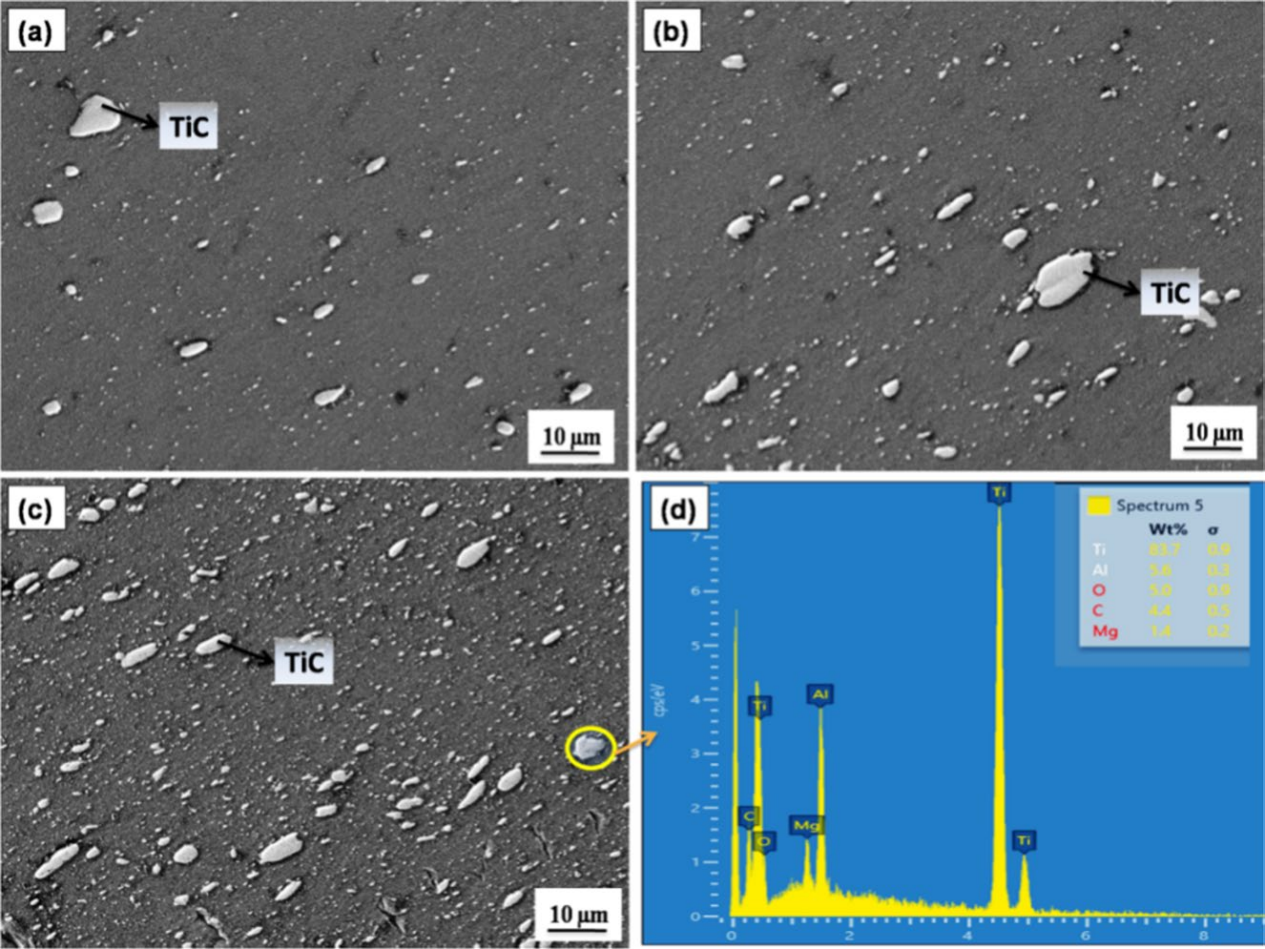
SEM images of the FSP composite specimens, specifically (**a**) 5 vol%, (**b**) 10 vol%, (**c**) AZ31 alloy with 15 vol% TiC and (**d**) EDS outcomes for 15 vol% TiC composites.

The SEM images of the AZ31 composites reinforced with 5 vol.%, 10 vol.% and 15 vol.% TiC are presented in Fig. 2(a–c). There are no visible flaws or microporosity in the micrographs of all the samples. The TiC particles are uniformly distributed throughout the matrix and exhibit strong interfacial bonding with the AZ31 alloy. The EDS mapping result for the AZ31/15 vol.% TiC composite is shown in Fig. 2(d).The elemental distribution maps clearly confirm the successful incorporation of TiC particles into the matrix, illustrating the spatial distribution of Mg, Zn, C and Ti within the analyzed area. “Furthermore, the AZ31/15 vol.% TiC composite exhibits a significantly refined grain structure, with an average grain size of approximately 8 µm, as reported in a previous study [ 30], compared to the coarser grain size of about 60 µm observed in the unreinforced AZ31 alloy. This type of structure can be attributed to dynamic recrystallization encouraged during FSP. The presence TiC particles are found to actively prevent grain growth, therefore improving the general microstructural features of the composite.

### Hardness and wear test results

Table 2 summarizes the hardness test findings for the AZ31 alloy and its composites. Higher TiC concentration clearly shows a progressive rise in the hardness of the composites. With a measurement of 116 ± 2.1 HV, the AZ31/15 vol% TiC composite shows the highest hardness value. When compared to the unreinforced AZ31 alloy**(**62 ± 1.2 HV), the rises in hardness for the AZ31/5TiC, AZ31/10TiC and AZ31/15TiC composites are about 27%, 59% and 83%, respectively. The intrinsic high hardness of TiC particles, which can withstand localized deformation under indentation, mostly explains the increase in hardness. The ceramic particles also serve as an efficient barrier to dislocation movement inside the matrix, therefore helping to increase the hardness of the composite^31^.

**Table 2:** Hardness measurements of the AZ31/TiC FSP composite specimens.

Material	Hardness (HV)
AZ31 alloy	62 ± 1.2
AZ31/5 vol% TiC	79 ± 1.6
AZ31/10 vol% TiC	99 ± 1.9
AZ31/15 vol% TiC	116 ± 2.1

Microstructural changes brought on by the addition of small TiC particles mostly explain the improvement in mechanical qualities. Orowan strengthening is important since the uniformly distributed TiC particles across the AZ31 matrix function as efficient barriers blocking dislocation movement. Dislocations in the composites result from the mismatch in thermal expansion coefficients between the TiC particles and the AZ31 alloy, as well as from differential deformation behavior during processing. Higher concentrations of reinforcement particles increase the barriers to dislocation movement, therefore producing a positive correlation between the TiC volume fraction and the degree of dislocation blocking. Moreover, the good interfacial bonding between the TiC particles and the AZ31 matrix promotes efficient load transfer, thereby improving the mechanical performance of the composite. The enhancement in mechanical characteristics is aided by the efficient transfer of stress from the ductile AZ31 matrix to the stiffer TiC particles.

**Fig. 3:**
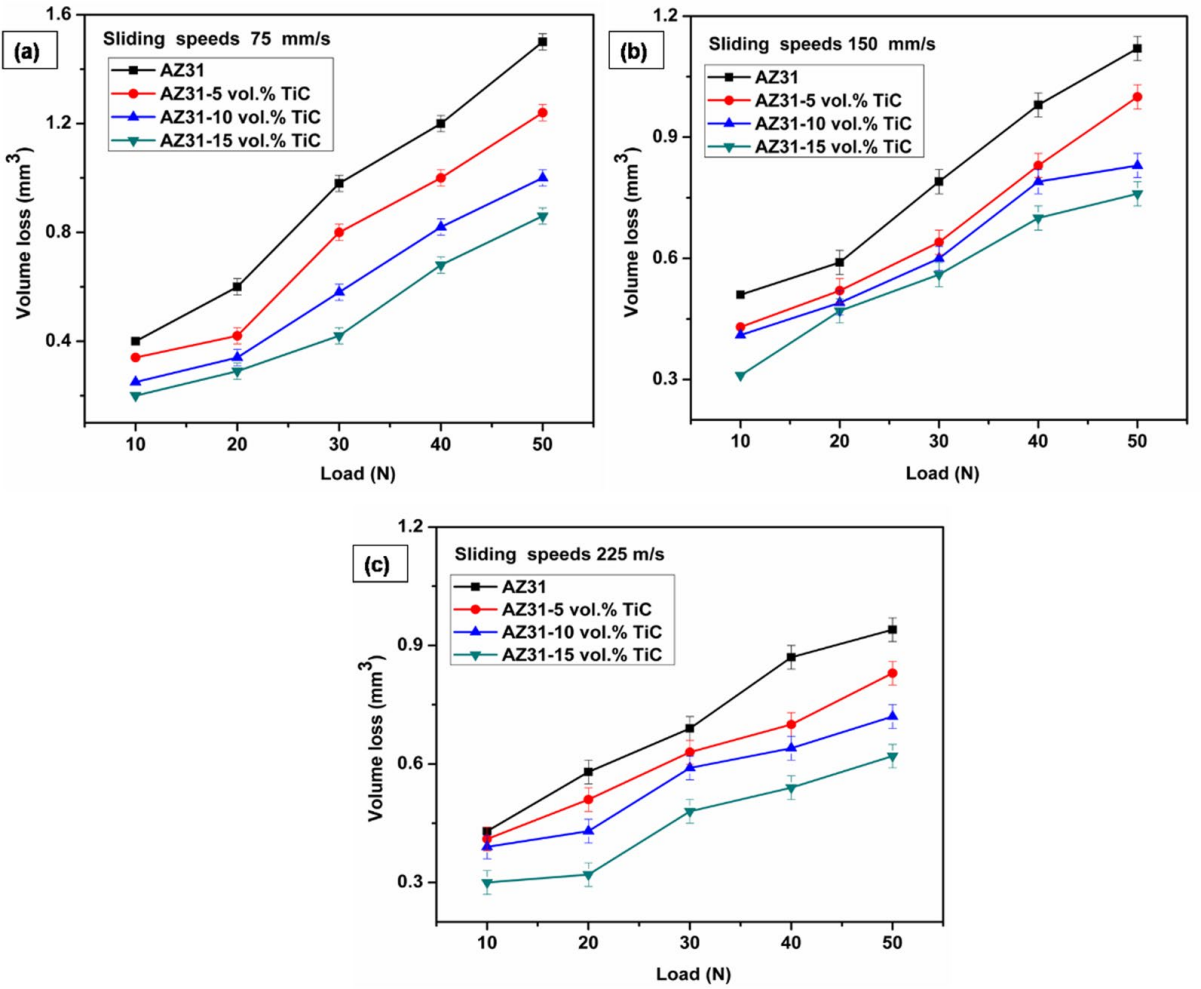
Volume loss distributions for composites under various sliding speeds (**a**) 75 mm/s, (**b**) 150 mm/s and (**c**) 225 mm/s. Error bars represent average of three trials (within ± 0.02 SD).

TiC particles also help to significantly refine the grains in the AZ31 matrix, thereby producing smaller sized grains. In line with the Hall-Petch relationship, which links decreased grain size with higher hardness and strength, this decrease in grain size improves the tensile stress resistance of the composite. These strengthening mechanisms grow more pronounced as the TiC volume fraction rises, which lowers the interparticle spacing and therefore limits dislocation mobility. Furthermore, more plastic deformation during processing causes a greater dislocation density, which is also quite important for the microhardness improvement of the composites. Therefore, the mechanical performance of the AZ31/TiC composites gets much better with increasing the TiC content. The reduction in volume loss as a function of load at different sliding speeds is shown in Fig. 3(a–c) for composites containing 5 to 15 vol% TiC. It is clearly seen that the volume loss diminishes as the TiC content increases, regardless of the sliding speeds and load. The lowest volumetric loss among all the tested samples showed that the AZ31/15 vol% TiC composite had the greatest wear resistance. Under a 50 N load and a 75 mm/s sliding speed, the unreinforced AZ31 alloy showed a volumetric loss of 1.45 mm³, which was lowered to 1.0 mm³ after the addition of 15 vol% TiC particles. At a sliding speed of 150 mm/s and under the same load, the AZ31/15 vol% TiC composite showed a volumetric loss of 0.62 mm³, or 34% less than the unreinforced AZ31 alloy, which reported a loss of 0.94 mm³.

Extensive studies have been published on the better wear performance of metal matrix composites, which is ascribed to several mechanisms. The great hardness of the reinforcement particles helps to increase the wear damage resistance of the material^32,33^. Moreover, the reinforcement particles are efficient load-bearing components, so lowering the stress carried by the softer matrix phase^34^. Minimizing material removal during sliding wear by means of the actual contact area between the metallic matrix and the steel counterface helps to improve tribological performance even more. As demonstrated in Fig. 3a–c, the volumetric loss observed in all samples escalates in correlation with the applied load. Reports indicate that wear performance deteriorates as the load increases due to greater deformation, which leads to higher contact pressure and an expanded contact area between the surfaces^35^. Additionally, as noted by Selvam et al.^36^, a higher applied load results in an increased wear rate due to the intensified ploughing effect.

Several studies in the literature have established a positive correlation between load and wear rate. Banijamali et al.^37^ and Turan et al.^38^ elucidated that magnesium-based alloys and composites demonstrate a substantial increase in wear rate accompanied with increase in loads. While Turan et al.^38^ found a comparable trend in Mg/MWCNT nanocomposites (0.25 and 0.5 wt%) under load conditions spanning 10 N to 40 N, Banijamali et al.^37^ observed a similar phenomenon in ZK60 and ZK60/3 wt% Y alloys under applied loads of 5, 20, 40 and 60 N. Figure 3(a–c) illustrates the relationship between the volumetric loss of composites containing 5 to 15 vol% TiC at various sliding speeds and loads. Irrespective of the applied load, the graphs clearly demonstrate that the volumetric loss decreases with increasing sliding speed for all composites. For example, under a load of 40 N, the AZ31/15 vol% TiC composite exhibited volumetric losses of 0.61 mm³, 0.58 mm³ and 0.56 mm³ at sliding speeds of 75 mm/s, 150 mm/s and 225 mm/s, respectively. However, a deviation from this general trend is observed at lower loads of 10 N and 20 N, where the volumetric loss slightly increased from 75 mm/s to 150 mm/s, suggesting that a different wear mechanism may dominate under these conditions.

Except for the AZ31 alloy under a load of 10 N, the volume loss of all composites is observed to decrease at a sliding speed of 150 mm/s. An increase in sliding speed enhances the strain hardening effect on the pin surface, thereby increasing the surface hardness and reducing the real contact area, which in turn improves the materials wear resistance^38^. Similar observations have been reported in several studies. Shen et al.^40^ and Shanthi et al.^41^ both documented a decrease in wear rates with increasing sliding speeds in AZ31-based composites. Specifically, Shen et al.^40^ investigated the wear behavior of AZ31 and AZ31/SiC (1 vol%) nanocomposites under applied loads of 10, 20 and 30 N at sliding speeds of 0.1, 0.2 and 0.5 m/s and found a significant reduction in wear rate with increasing sliding speed. Similarly, Shanthi et al.^41^ reported that the wear rate of AZ31/Al₂O₃ composites decreased as the sliding speed increased from 1 m/s to 10 m/s.

**Fig. 4:**
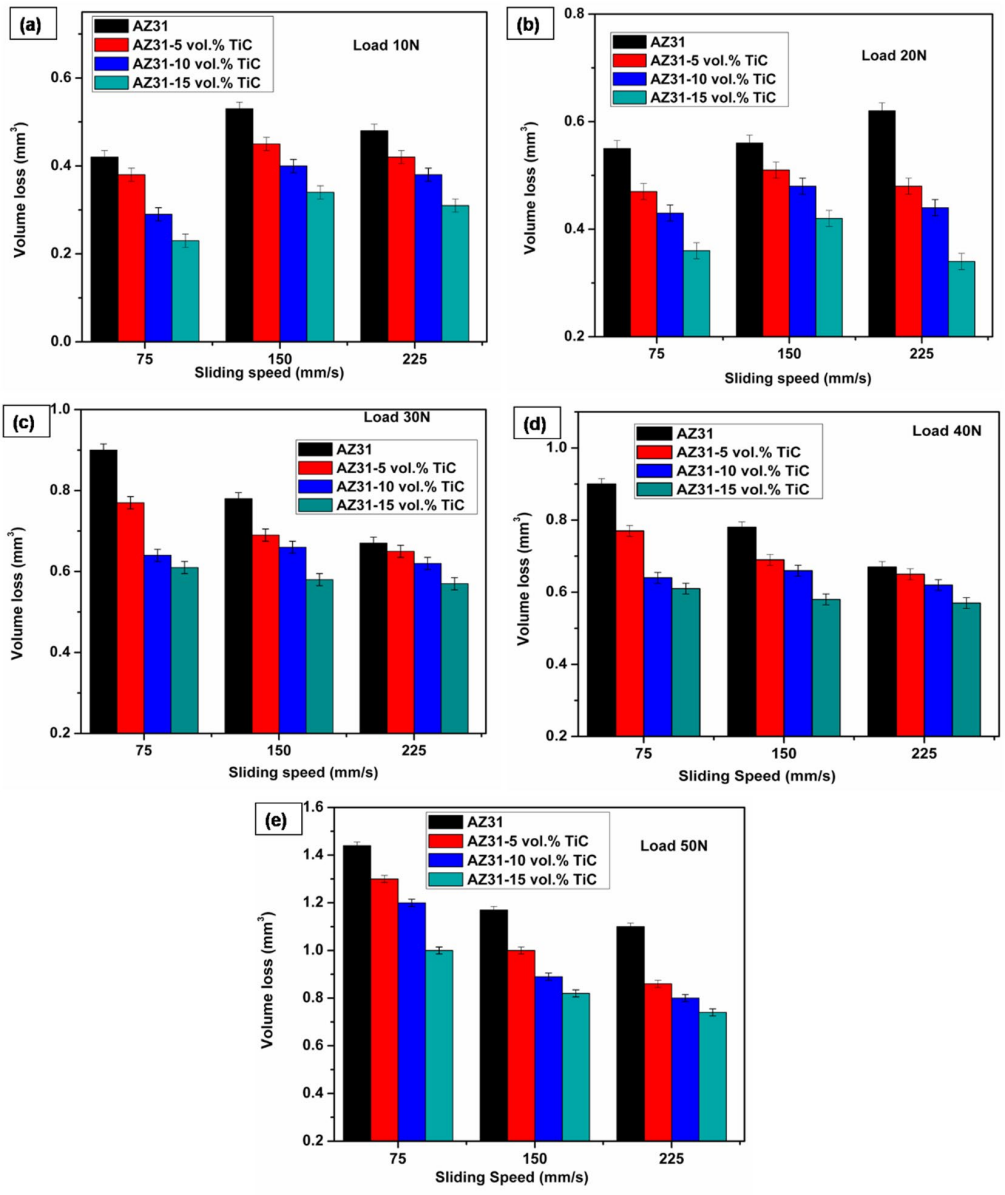
Volume loss as a function of sliding speed for different loads (**a**) 10 N, (**b**) 20 N, (**c**) 30 N, (**d**) 40 N and (**e**) 50 N. Error bars represent average of three trials (within ± 0.02 SD).

At lower speeds due to micro-cutting mechanisms more material removal occurs, leading to such behaviour. Researchers have also linked this trend to the major impact of friction-induced thermal effects between the matrix and counterface, therefore affecting the general wear process. Abrasive interactions between surfaces of varying hardness produce a higher friction coefficient at lower sliding speeds. At greater sliding speeds, the rise in contact temperature encourages surface oxidation, causing a protective oxide layer formation. These layers lower the wear rate as well as the friction coefficient. The results of Aung et al.^42^ under low-stress conditions match those of the current study, therefore supporting the noted wear behavior. The impact of the TiC content on the wear behavior is also evident in Fig. 4a-e. The volume loss is consistently found to decrease across all applied loads and sliding speeds as the TiC concentration increases. Under a load of 10 N and a sliding speed of 75 mm/s, the AZ31/15 vol% TiC composite showed a 54.7% drop in volume loss (0.23 mm³) relative to the AZ31 alloy (0.42 mm³). At a greater load of 50 N and the same sliding speed, the AZ31 alloy also recorded a volume loss of 1.5 mm³. Composite including 15 vol% TiC showed a far lower volume loss of 0.86 mm³. These results show that, regardless of the size of the applied load, TiC particles significantly increase the wear resistance of the composites.

Improved hardness and the existence of TiC reinforcements mostly explain the better wear resistance of the composites. These findings support Archard’s law, which holds that materials with greater hardness usually show better tribological performance^43^. Many studies have likewise found that the inclusion of ceramic reinforcements improves the wear resistance of metal matrix composites. Behnamian et al.^8^, for instance, studied the tribological behaviour of ZK60/B_4_C/MWCNT hybrid composites and reported that a higher MWCNT concentration resulted in a lower wear rate. Owing to the self-lubricating characteristics of multi-walled carbon nanotubes (MWCNTs), this improvement is ascribed to a drop in the coefficient of friction (COF).

Similarly, Thirugnanasambandham et al.^44^ investigated the wear properties of AZ91/SiC composites under sliding speeds of 0.25, 0.5 and 0.75 m/s and loads varying from 10 to 40 N. Their findings showed that the addition of SiC particles improved the wear resistance under all examined circumstances. Specifically, at a load of 40 N and a sliding velocity of 0.75 m/s, the wear rates for specimens with 0, 2.5, 5 and 7 wt% SiC were reported as 0.260, 0.250, 0.240 and 0.175 mg/m, respectively. Likewise, Lim et al.^45^ looked at the tribological performance of Mg/Al₂O₃ composites with different volume fractions (0.22, 0.66 and 1.11 vol%) and found that the composite with 1.11 vol% Al₂O₃ showed the greatest wear resistance. Consistent with these studies, the current research shows a distinct rise in the wear performance of AZ31/TiC composites with an increase in reinforcement material. The agreement of these results with earlier published data confirms even more the role of ceramic reinforcements in improving the tribological characteristics of magnesium-based composites.

**Fig. 5:**
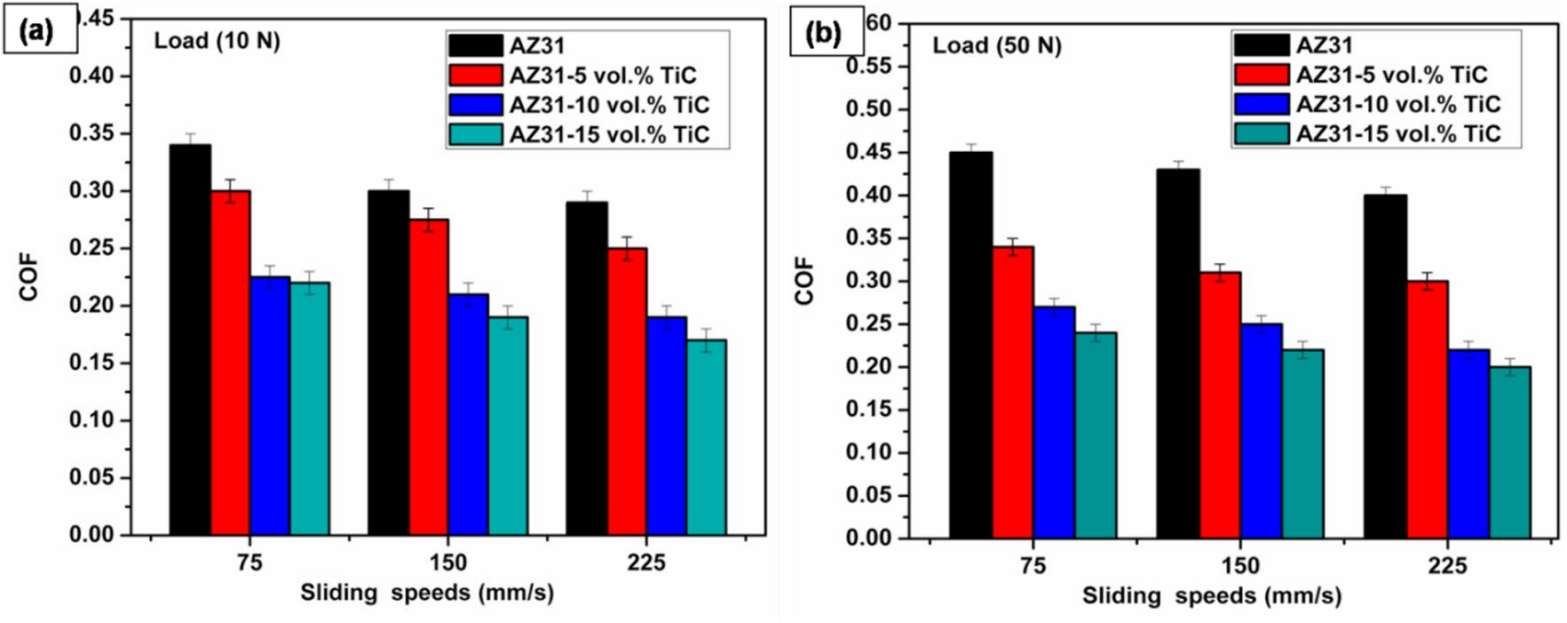
Coefficient of friction (COF) variations for AZ31 and composites under loads of (**a**) 10 N and (**b**) 50 N at varying sliding speeds. Error bars represent average of three trials (within ± 0.02 SD).

Measured under applied loads of 10 and 50 N and at sliding velocities of 75, 150 and 225 mm/s over a constant sliding distance of 1500 m, the COF for AZ31, AZ31/5 vol% TiC, AZ31/10 vol% TiC and AZ31/15 vol% TiC composites are presented in Fig. 4. The findings elucidate a reduction in COF concomitant with an increase in TiC vol% and sliding velocity, whereas an augmented applied load results in an elevation of COF across all specimens. The mean COF for the AZ31/15 vol% TiC composite at sliding velocities of 75, 150 and 225 mm/s is approximately 0.22, 0.19 and 0.17 under a 10 N load (Fig. 5a) and 0.24, 0.22 and 0.20 under a 50 N load (Fig. 5b). An increase in reinforcement content enhances the load-bearing capability of the composite and mitigates direct contact within the magnesium matrix. As the composite surface shows more resistance to frictional forces^45^, the higher energy needed for shearing the reinforcement particles lowers the COF. The reinforcement particles also limit plastic deformation, therefore helping to lower the friction between the interacting surfaces^46^. The inclusion of reinforcement particles has been linked in several studies to a drop in COF^47^. Behnamian et al.^8^, for example, found that the COF of ZK60/10B_4_C/MWCNT composites dropped with more MWCNT content, averaging COF values of 0.21, 0.20 and 0.18 for MWCNT concentrations of 0%, 0.1% and 0.5%, respectively, under a 40 N load. The development of a carbon coating on the surface of the MWCNTs, which functioned as a solid lubricant, explained this drop. Zhu et al.^48^ likewise noted that the COF of Mg/SiC/WS₂ hybrid composites fell with rising SiC concentration, which was ascribed to a reduction in interfacial frictional forces.

As shown in Fig. 5, a clear correlation exists between COF and load, regardless of sliding speed or material composition. The increase in COF with higher loads aligns with previous studies^45,46^ and is primarily due to greater plastic deformation. Furthermore, this investigation substantiates a reduction in the coefficient of friction (COF) with the augmentation of sliding velocity. Existing literature posits that the escalation in wear rate at increased speeds can be attributed to the relentless removal of oxides from the sample surface. Elevated sliding velocities facilitate the detachment of the worn surface and the emergence of delaminations, consequently resulting in a diminished friction coefficient. Additionally, reports indicate that increased sliding speed facilitates the formation of an oxide layer on the metallic surface due to oxygen diffusion, further reducing friction^45^.

### Surface examination of wear

**Fig. 6:**
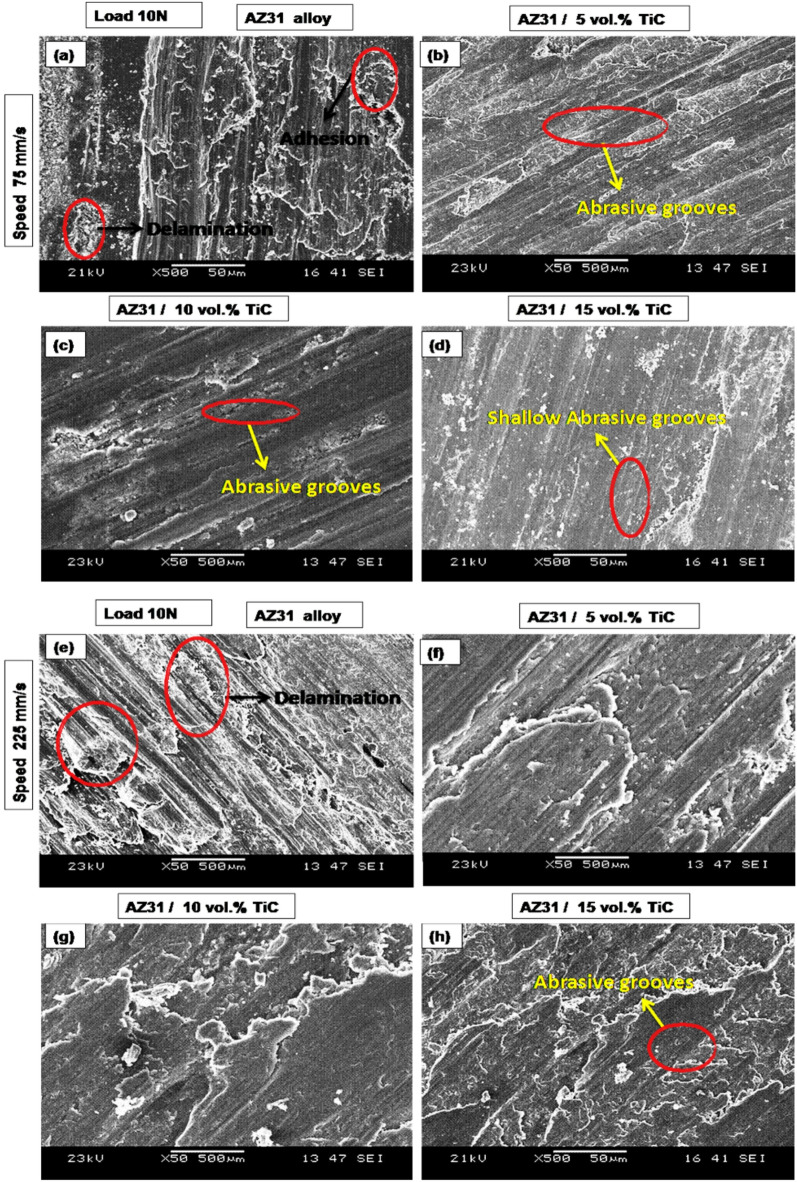
SEM micrographs of AZ31 alloy and AZ31/TiC composites under a load of 10 N at two sliding speeds: (**a**–**d**) 75 mm/s and (**e**–**h**) 225 mm/s. The microstructures correspond to (**a**,** e**) AZ31 alloy, (**b**,** f**) AZ31/5 vol% TiC, (**c**,** g**) AZ31/10 vol% TiC and (**d**,** h**) AZ31/15 vol% TiC.

Figure 6(a-e) presents SEM images of the samples evaluated at a sliding speed of 75 mm/s (Fig. 6(a–d)) and 225 mm/s (Fig. 6(e–h)) under a 10 N load. The profound indentations and particles resulting from friction were observed at 10 N load (Fig. 6a). When the sliding speed is increased, the AZ31 alloy exhibited a significant delaminated region and several adhesions, as seen in Fig. 6e. Delamination wear occurs when a significant load causes shear deformation, resulting in the production of cracks^49^. Behnamian et al.^8^ found that the primary wear process for ZK60 / MWCNTs / B_4_C hybrid composites under high stress (80 N) was delamination and oxidation. Figure 6f-h shows the presence of abrasive grooves on the worn surface of the AZ31/ 5, 10 and 15 vol% TiC composites. Examining the worn surfaces of samples under a load of 10 N and sliding speed of 75 mm/s shows that higher TiC concentration transforms broad abrasive grooves into finer scratches. This change shows a decrease in wear-related damage and therefore an increase in wear resistance. Among the assessed samples, the AZ31/15 vol% TiC composite showed the least wear damage, supporting the results shown in the volume loss graph (Fig. 4).

**Fig. 7:**
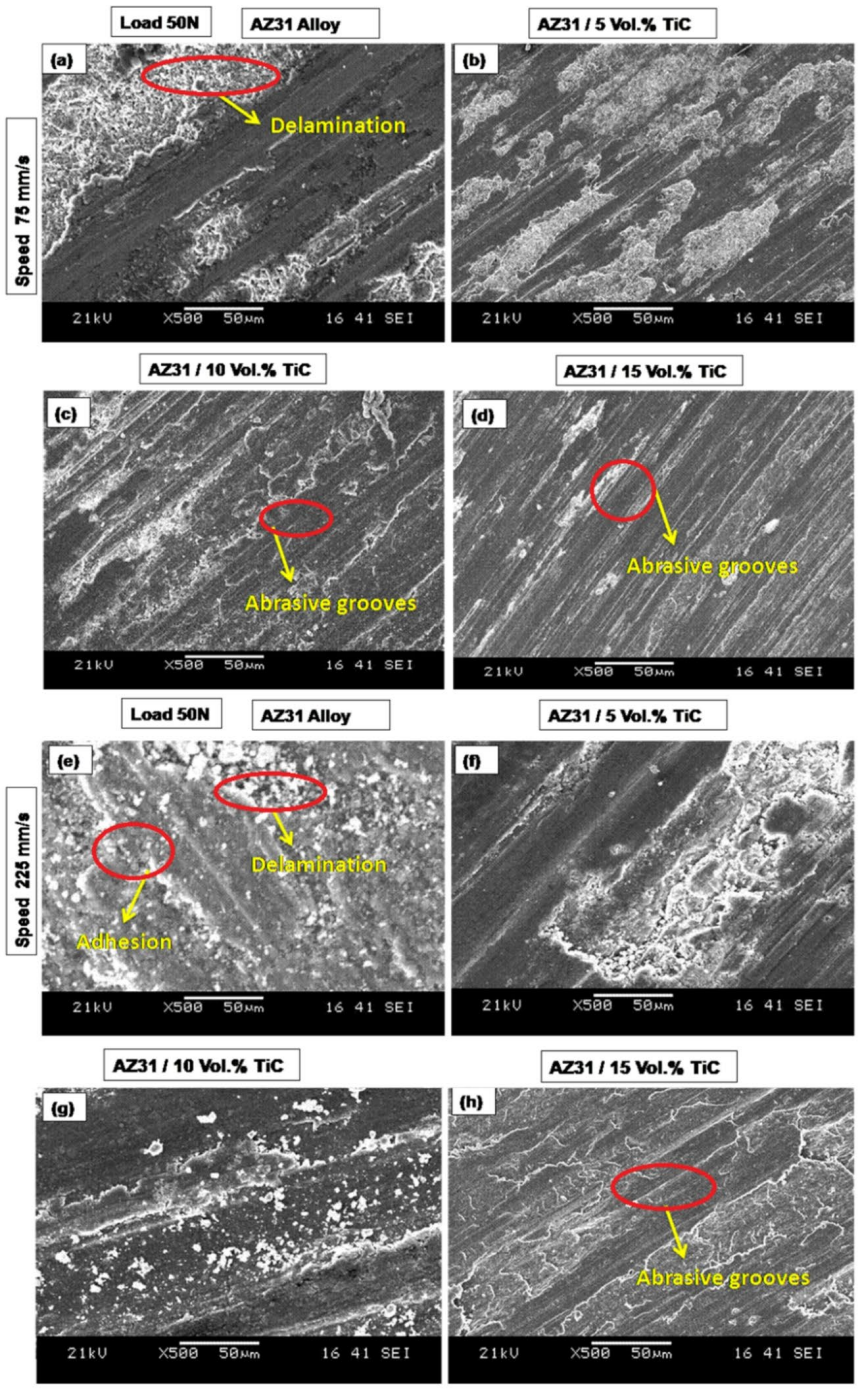
SEM micrographs of AZ31 alloy and AZ31/TiC composites under a load of 50 N at two sliding speeds: (**a**–**d**) 75 mm/s and (**e**–**h**) 225 mm/s. The microstructures correspond to (**a**,** e**) AZ31 alloy, (**b**,** f**) AZ31/5 vol% TiC, (**c**,** g**) AZ31/10 vol% TiC and (**d**,** h**) AZ31/15 vol% TiC.

Under a 50 N load, Fig. 6 shows SEM images of the worn surfaces of the specimen evaluated at a sliding speed of 75 mm/s (Figs. 7a–d) and 225 mm/s (Figs. 7e–h). The worn surface of the AZ31 alloy shows clear delamination and adhesive wear as seen in Fig. 6a. Distinct fissures appear under a 50 N applied load, suggesting that the wear mechanism changes into a more severe delamination process. This change from mild to severe wear fits earlier results^8^. Furthermore, (Fig. 7e), crater formation becomes clear on the AZ31 alloy surface as the sliding speed rises from 75 mm/s to 225 mm/s. It can be concluded that Delamination is responsible for formation of the craters on the sample surface.

The worn surface of the AZ31/5 vol% TiC composite exhibits grooves under a 10 N load, whereas multiple cracks and craters become prominent at a 50 N load (Fig. 7c–d). It is well established that an increase in applied load promotes delamination and plastic deformation^50^. Additionally, the presence of fine scratches on the worn surface of AZ31/10 vol% TiC suggests a moderate abrasive wear mechanism (Fig. 6e), further confirming the role of TiC reinforcement in enhancing wear resistance.

When subjected to increased stress, the grooves and particles resulting from wear were observed, indicating the occurrence of abrasive wear (Fig. 7f). The AZ31 / 15 TiC may exhibit a smooth surface with apparent scratches. Furthermore, it is seen that the moderate grooves persist even under high stress conditions, as depicted in Fig. 7h.

**Fig. 8:**
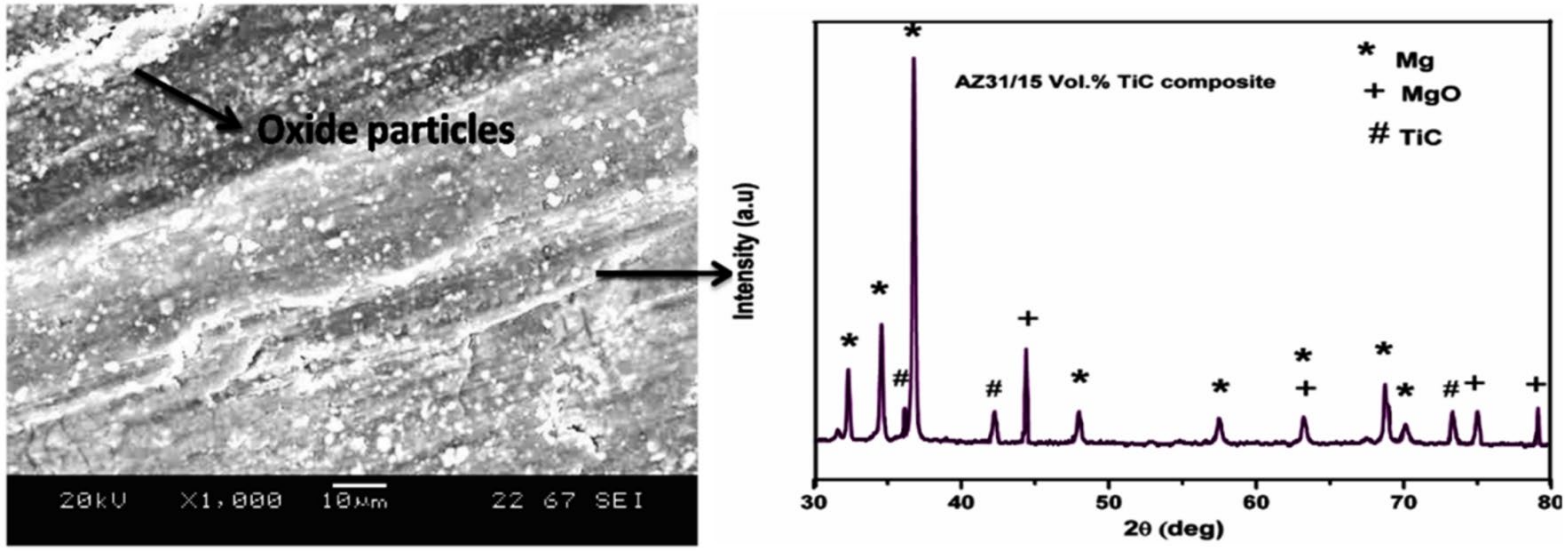
XRD results of AZ31 / 10 vol% TiC subjected to a load of 50 N while sliding at a speed of 225 mm/s.

Figure 8 presents the XRD analysis of the AZ31/10 vol% TiC composite subjected to a 50 N load at a sliding speed of 225 mm/s. The XRD study reveals that a substantial concentration of MgO is formed due to the intense friction generated at a high load of 50 N. Consequently, it can be deduced that the eroded surfaces are enveloped by an oxide layer. According to extant literature, MgO is generated because of abrasive wear and subsequently experiences fracturing during the experimental evaluation process^8^. Numerous scholars have substantiated the existence of increased oxygen concentrations on the abraded surfaces of magnesium matrix composites via EDS and XRD analytical techniques, signifying the development of oxidation layers that significantly affect the wear characteristics.

## ML-Based prediction of volume loss

This study presents an experimental approach developing a predictive mapping between TiC content, sliding speed and applied load as input parameters and volume loss as output parameters, using multiple supervised ML regression models. Initially, the experimental data was systematically organized and preprocessed to ensure its suitability for ML applications. The dataset was then partitioned into two subsets: training data (80%) and validation data (20%). Five distinct ML models were developed and trained using the training dataset.

In total 135 wear measurements were collected, corresponding to 45 unique test conditions (combinations of three TiC levels, five loads and three speeds) each repeated in triplicate to quantify measurement variability. Condition‑wise means and standard deviations were computed and used for model fitting and replicates belonging to the same condition were kept together during cross‑validation using a Group K Fold strategy. Only three controllable, a‑priori factors—TiC content, load and sliding speed—were used as predictors. Although microstructural attributes such as grain size, hardness and dislocation density were measured experimentally, they represent downstream consequences of the processing conditions and would introduce information leakage and multicollinearity into the models. A pilot ablation study including hardness and grain size confirmed that these additions did not improve cross‑validated accuracy and instead increased variance. We therefore restricted the model inputs to the controllable factors and emphasize that the reported high R² values arise from interpolation within the factorial design space rather than extrapolation beyond it.

Data preparation and model evaluation were performed using a leakage‑free pipeline: (1) an 80/20 train–test split was made on the condition‑wise means; (2) a scikit‑learn Pipeline was constructed chaining StandardScaler and each estimator; (3) GridSearchCV with 5‑fold cross‑validation was applied on the training set only, ensuring that scaling and model fitting occurred inside each fold; (4) the best hyperparameters were selected based on the mean cross‑validated R² and the final model was evaluated on the untouched 20% test set. This procedure prevents optimistic bias due to data leakage.

To probe robustness beyond simple random splits, we also conducted leave‑one‑factor‑level‑out cross‑validation (e.g., excluding all data at 50 N during training) and grouped folds by FSP stir zone location. Predictive accuracy decreased under these more stringent protocols—particularly when the highest load level was held out—but the relative ranking of algorithms (RF ≥ GB ≫ LR) remained unchanged. These additional analyses suggests that the developed models interpolate reliably within the studied domain while highlighting that true external validation requires additional FSP batches or different counterface materials, which are planned for future work.

To ensure model robustness, a 5-fold cross-validation technique was employed, wherein the training dataset was divided into five subsets, with each subset iteratively used for validation. This step ensured that the models were assessed for generalizability and overfitting before final evaluation. To determine the optimal model configuration, a grid search method was employed, systematically testing multiple hyperparameter configurations for each model. Cross-validation was performed to identify the best-performing model based on training data. The most accurate models were subsequently evaluated on the validation dataset and the results are discussed in this section. The ML dataset comprised 135 individual wear measurements derived from 45 unique experimental conditions (three TiC contents × five loads × three speeds) with three repeats per condition; unless otherwise noted, condition‑wise means were used as input to the models. This approach acknowledges the limited sample size and reduces variance by pooling replicates.

### ML models

**Fig. 9:**
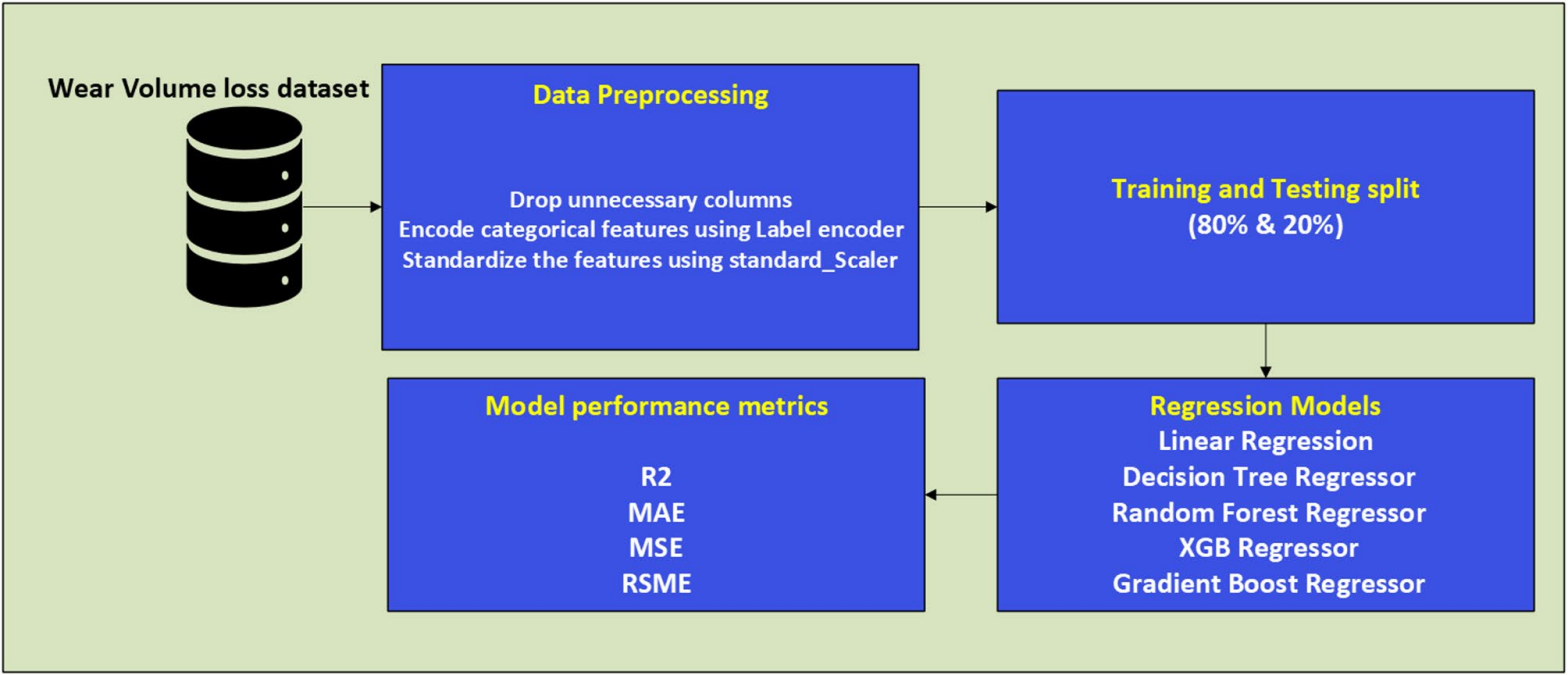
Comprehensive block diagram illustrating the stages needed in ML.

Five widely used regression models (Linear Regression (LR), Decision Tree (DT), Random Forest (RF), Gradient Boosting (GB) and Extreme Gradient Boosting (XGB)) were selected for performance comparison on the given dataset. The application of regression methodologies was necessitated by the nature of the dependent variable—volume loss—which is characterized as a continuous real-valued metric. The execution of all model implementations was conducted utilizing the Python programming language, with the computational tasks executed on the Google Colab platform. The scikit-learn package was utilized for ML tasks, while Seaborn was employed for data visualization. To enhance prediction accuracy, data preprocessing was performed using the Standard Scaling algorithm, which normalizes input and output features to improve the efficiency of ML models. Standardization ensured consistency across varying data scales, leading to more precise and reliable predictions. A detailed block diagram depicting the essential stages of the ML process is shown in Fig. 9. A pseudocode of the entire process is also shown as Algorithm 1.

**Algorithm 1:**
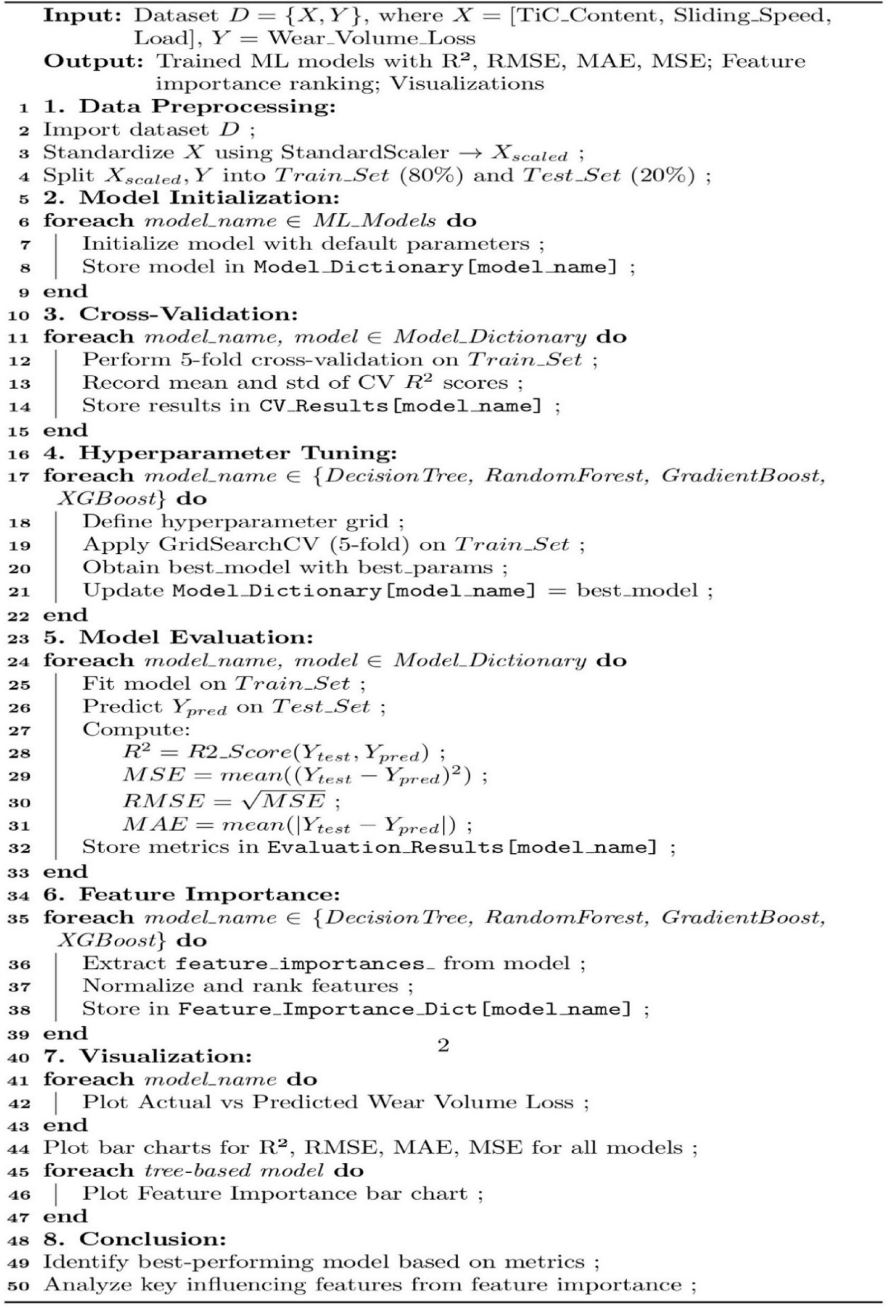
ML- based wear volume loss prediction and feature analysis.

#### Linear regression

By creating links between dependent and independent variables, ML—especially the linear regression method—fundamentally drives predictive modeling. A supervised learning algorithm called linear regression minimizes the error between actual and predicted values to fit a linear equation to a given dataset. Its simplicity and interpretability make this approach popular in many sectors, including engineering, economics and healthcare. Usually, model parameters are estimated by means of the least squares technique, which reduces the sum of squared residuals. Though it is good in modeling linear connections, linear regression has significant drawbacks when dealing with complicated, nonlinear data, so requiring the use of sophisticated ML methods like polynomial regression or neural networks. Still, linear regression is a basic approach in data-driven decision-making since it offers insightful analysis of trends and patterns across many uses.

#### Decision tree (DT)

The decision tree model predicts output values depending on the input features by means of a tree-like structure made up of nodes and leaves. Decisions at every node are made according to splitting criteria derived from feature values, such as information gain, Gini impurity, or mean squared error (MSE). Every leaf node is a last prediction matching a numerical target value in regression work. The criterion used to assess the efficacy of every split is the main driver of decision-making in decision trees. Node partitioning is often done using metrics like Mean Absolute Error (MAE) and Mean Squared Error (MSE). A more complicated model may result from deepening the tree, which could cause overfitting where there is a situation in which the model remembers the training data and underperforms on unobserved test data.

#### Random forest (RF)

Random Forest (RF), a supervised ML method, offers better performance in regression and classification activities. It builds several decision trees from randomly chosen training data portions. Every tree is trained separately in regression jobs and the last prediction comes from averaging the results of all trees. The number of trees in the forest is defined by *n_estimators* which is a main hyperparameter. Inherited from the Decision Tree model, other parameters are minimum samples per split and maximum depth. By improving model accuracy and reducing overfitting, the ensemble method confirms Random Forest as a strong and consistent predictive modeling tool.

#### Gradient boosting regressor

A very efficient ensemble ML technique, gradient boosting builds models sequentially, each following one trying to fix the errors of its predecessor. Its remarkable predictive accuracy and ability to manage complicated and high-dimensional data make it especially popular for regression tasks. This approach iteratively combines multiple weak learners into a robust predictive model. The basic idea is to gradually add new models forecasting the residuals (errors) of the combined ensemble from prior iterations, therefore minimizing a differentiable loss function. Gradient Boosting methodically increases the performance by concentrating on the errors at every stage, therefore enabling it to catch complex patterns in the data. Inspite of its benefits, hyperparameter tuning including tree depth, number of estimators and learning rate is crucial to avoid overfitting and guarantee best model generalization. Given a dataset D={(x_i_, y_i_)} ^n^i=1, where denotes the feature vector and represents the corresponding target value, the goal is to identify a model () that minimizes the loss function (y, F(x)). Through this iterative approach, Gradient Boosting enhances the model’s accuracy by concentrating on the data points that previous models struggled with, leading to a strong predictive model that can tackle complex regression challenges.

#### Extreme gradient boosting (XGB)

Specifically meant to forecast output variables from input features, Extreme Gradient Boosting (XGB) is a sophisticated, tree-based supervised ML technique. Unlike Random Forest, which constructs trees independently and in parallel, XGB constructs trees sequentially, each new tree rectifying the mistakes of the prior ones. High predictive performance results from this boosting technique, which makes XGB especially good at managing structured and tabular data for regression and classification tasks. Like RF, XGB consists of multiple decision trees, but its training mechanism is fundamentally different. Additionally, XGB incorporates an L2 regularization function to control model complexity, reducing overfitting and enhancing generalization. This makes XGB a more efficient and precise alternative to traditional Gradient Boosting models.

### Hyper parameter optimization

Hyperparameter optimization was performed using the Grid Search CV method from the scikit-learn library, employing a 5-fold cross-validation strategy for each model to identify the optimal combination of parameters. The search grids were designed to balance model performance with computational efficiency, particularly considering the limited size of the dataset. The hyperparameters and their respective ranges explored for each model are as given in Table 3.

**Table 3:** Hyperparameters and their tuning ranges.

Model	Hyperparameter	Values
Decision Tree	max_depth	3, 5, 10, None
	min_samples_split	2, 5, 10
Random Forest	n_estimators	50, 100, 200
	max_depth	5, 10, None
	min_samples_split	2, 5, 10
Gradient Boost	n_estimators	50, 100, 200
	learning_rate	0.01, 0.1, 0.2
	max_depth	3, 5, 10
XGBoost	n_estimators	50, 100, 200
	max_depth	3, 5, 10
	learning_rate	0.01, 0.1, 0.2

Each model was evaluated using R², RMSE, MAE and MSE on the validation folds. The final model configurations were selected based on the highest mean R² score across folds. This systematic tuning ensures that all models were fairly optimized for their respective parameter spaces and prevents performance bias due to arbitrary parameter selection.

The parameter tuning phase has been initiated to ascertain the optimal configuration for each ML model. Owing to the discrepancies in tuning configurations for each model, the combinations of parameter subsets have been tailored individually.

Upon completion of the hyperparameter optimization phase, the ideal configuration for each model is determined. The ideal configuration for the Random Forest (RF) model is as follows: the maximum number of features considered for the best split (MF) is established at 2, the minimum number of samples necessary at a leaf node (MSL) is set to 1 and the total number of decision trees in the forest (NE) is fixed at 100. An optimal configuration for XGB consists of a learning rate (η) of 0.1 and an estimator of 50 trees. The optimal design of the decision tree involves using the absolute error as the splitting criterion, setting the maximum depth level to 8 and the minimum sample leaf (MSL) to 1.

As mentioned earlier, four distinct measures, namely R^2^, RMSE, MSE and MAE, were utilized in this work to evaluate and compare the performances of the ML models. The coefficient of determination, R^2^, is a statistical concept used to assess the goodness of fit of a regression function. It is derived using the following Eq. (1).

1 $$R^{2} = \frac{{\sum\limits_{{i = 1}}^{n} {\left( {y_{i} - \hat{y}_{i} } \right)^{2} } }}{{\sum\limits_{{i = 1}}^{n} {\left( {y_{i} - \bar{y}} \right)^{2} } }}$$

Let* n* denote the quantity of assessments, $\:yi$ signify the empirically measured output value, $\:\hat yi$ denote the anticipated output value and$\:y$ represent the arithmetic mean of the empirically measured values. The RMSE, or root mean squared error, serves as an indicator of the mean divergence between forecasted and actual values. It is computed by ascertaining the square root of the mean of the squared differences between the anticipated and actual values Eq. (2).

2 $$RMSE = \sqrt {MSE} = \sqrt {\frac{1}{n}} \sum\nolimits_{{i = 1}}^{n} {\left( {y_{i} - \bar{y}_{i} } \right)^{2} }$$

The MSE, or mean squared error, is a measure of the average squared difference between the actual and projected values. It is calculated using the following Eq. (3):

3 $$MSE = \frac{1}{n}\sum\limits_{{i = 1}}^{n} {\left( {y_{i} - \bar{y}_{i} } \right)^{2} }$$

The MAE, or mean absolute error, is a measure of the average difference between the actual and anticipated values. It is derived using the following Eq. (4):

4 $$MAE = \frac{1}{n}\sum\limits_{{i = 1}}^{n} {\left| {y_{i} - \bar{y}_{i} } \right|^{2} }$$

As shown in Eq. 1, the coefficient of determination (R²) evaluates how well the model’s predictions match the actual data. A perfectly fitting model has an R² value of 1, while a value of 0 indicates that the model fails to explain the variability in the data. R² is particularly effective in assessing the proportion of variance captured by the model compared to other metrics. Moreover, three supplementary error metrics serve to quantify the efficacy of the model: Root Mean Squared Error (RMSE) and Mean Squared Error (MSE) evaluate the squared deviations between actual and forecasted values. Given that RMSE represents the square root of MSE, both metrics exhibit a monotonic relationship—implying that reduced values signify enhanced model precision. Mean Absolute Error (MAE) computes the average absolute difference between actual and predicted values. Unlike RMSE and MSE, MAE is less sensitive to outliers, making it a useful metric when extreme deviations need to be minimized.

**Table 4:** Comparative performance of ML models without hyperparameters optimization.

ML Regressor	*R* ^2^	RMSE	MSE	MAE	CV Mean	CV SD
Linear Regression	0.7713	0.1166	0.0136	0.0792	0.7121	0.0370
Decision Tree	0.9945	0.0181	0.0003	0.0047	0.9856	0.0287
Random Forest	0.9919	0.0218	0.0005	0.0093	0.9827	0.0227
Gradient Boost	0.9952	0.0169	0.0003	0.0128	0.9866	0.0058
XGBoost	0.9967	0.0138	0.0002	0.0114	0.9896	0.0074

**Table 5:** Comparative performance of ML models with hyperparameters optimization.

ML Regressor	R2	RMSE	MSE	MAE	CV Mean	CV SD	Prediction Time (ms)	Memory Usage (MB)
Linear Regression	0.7713	0.1166	0.0136	0.0792	0.7121	0.0370	8.7 ± 0.5	62
Decision Tree	0.9945	0.0180	0.0003	0.0046	0.9855	0.0287	62	52
Random Forest	0.9919	0.0218	0.0004	0.0093	0.9827	0.0226	38	38
Gradient Boost	0.9987	0.0087	0.0007	0.0069	0.9940	0.0052	38	38
XGBoost	0.9963	0.0147	0.0002	0.0108	0.9902	0.0072	38.5	38.5

**Fig. 10:**
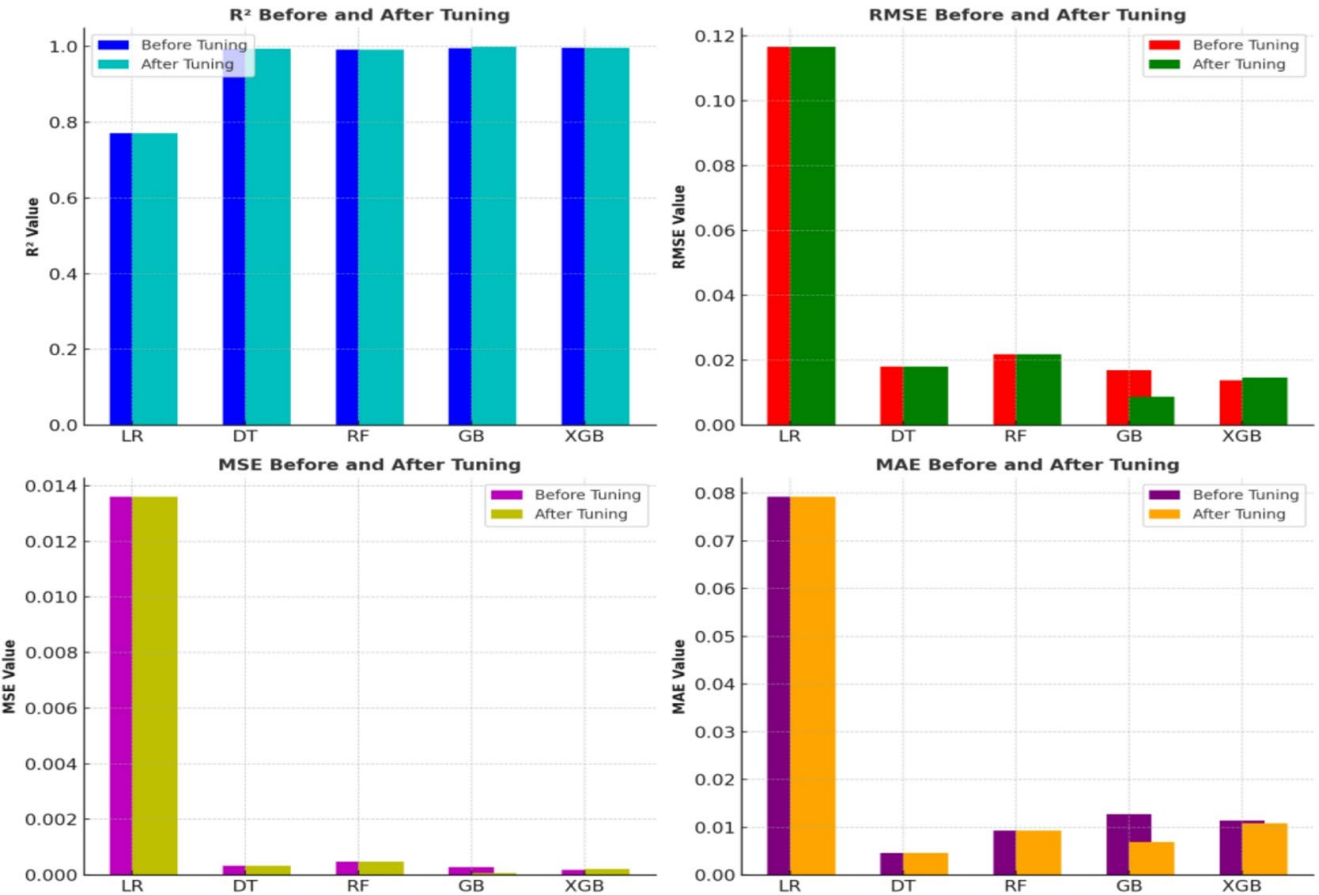
Comparative bar plots of R², RMSE, MSE and MAE before and after hyperparameter tuning.

The performance of machine learning models before and after hyperparameter tuning, as summarized in Tables 4 and 5, shows substantial improvements with tuning. The bar plots for R², RMSE, MSE and MAE (Fig. 10) highlight these enhancements. Among the models, Gradient Boost and XGBoost exhibited the most considerable improvements, with Gradient Boost achieving the highest R² = 0.9987 and the lowest RMSE = 0.087 after tuning. XGBoost also showed improvement, reaching R² = 0.9963 and RMSE = 0.0147 after tuning. In contrast, Linear Regression displayed no significant change in performance, as both the R² = 0.7713 and RMSE = 0.1166 remained unchanged before and after hyperparameter optimization, highlighting the model’s limitations with the given dataset. Although Gradient Boost showed a near-optimal R² of 0.9987 and RMSE of 0.0080, it is critical to evaluate the precision of these results relative to the inherent experimental measurement uncertainty. To validate these findings, the measurement uncertainty was estimated based on repeatability trials and the standard deviation of wear volume measurements across triplicates, yielding an estimated experimental uncertainty margin of ± 0.05 mm³. Accordingly, the RMSE values for both Gradient Boost and XGBoost (0.0087 and 0.0147, respectively) are well within this margin, indicating that the discrepancies between predicted and observed wear values are within the acceptable tolerance of the measurement system. This suggests that the model deviations are not physically significant and lie within the instrument’s error margin.

### Confidence interval analysis of R² scores

The performance of the machine learning models was evaluated using the coefficient of determination (R²) and 95% confidence intervals (CIs) from bootstrapped resampling as shown in Table 6. GB and XGBoost demonstrated the highest R² values of 0.9987 and 0.9963, respectively, indicating exceptional predictive performance on the training dataset. These models also exhibited slightly wider confidence intervals, suggesting that while they are highly accurate, there is moderate uncertainty in their ability to generalize to unseen data. In comparison, the Random Forest model also showed robust performance with an R² of 0.9919 and a narrow confidence interval (0.9969, 0.9996), suggesting robust and stable predictions across various conditions. The Decision Tree model, while achieving a high R² of 0.9945, had a broader confidence interval (0.9854, 0.9966), indicating potential challenges with overfitting and a risk of less reliable generalization. Finally, Linear Regression demonstrated significantly lower predictive performance with an R² of 0.7713, accompanied by a broad confidence interval, which highlights its limitations in capturing complex patterns within the dataset.

**Table 6:** Confidence interval analysis of R² Scores.

Model	*R*² Score	95% CI (Lower, Upper)
Linear Regression	0.7713	(0.6505, 0.8429)
Decision Tree	0.9945	(0.9854, 0.9966)
Random Forest	0.9919	(0.9969, 0.9996)
Gradient Boost	0.9987	(0.9922, 0.9988)
XGBoost	0.9963	(0.9882, 0.9980)

Comparative bar plots of R², RMSE, MSE and MAE before and after hyperparameter tuning are shown in Fig. 10. The R² values before and after hyperparameter tuning are consistently high across the models, with Gradient Boost and XGBoost maintaining the highest values, reflecting excellent predictive accuracy. The Random Forest model also shows robust performance, while LR exhibits the lowest R², indicating poor predictive power. RMSE, MSE and MAE: Hyperparameter tuning significantly improved the RMSE, MSE and MAE for GB and XGBoost, resulting in smaller values post-tuning, which suggests better model fit and less error in predictions. The DT and RF models show minor improvements, while Linear Regression’s performance remains unchanged, highlighting its limitations in capturing the underlying data patterns effectively. This plot and its analysis (Fig. 10) clearly demonstrate the effectiveness of hyperparameter tuning in enhancing model performance, particularly for GB and XGBoost, which exhibit notable improvements across multiple metrics. However, the inherent limitations of Linear Regression are still evident despite tuning, as it fails to show significant improvements in these key metrics.

**Fig. 11:**
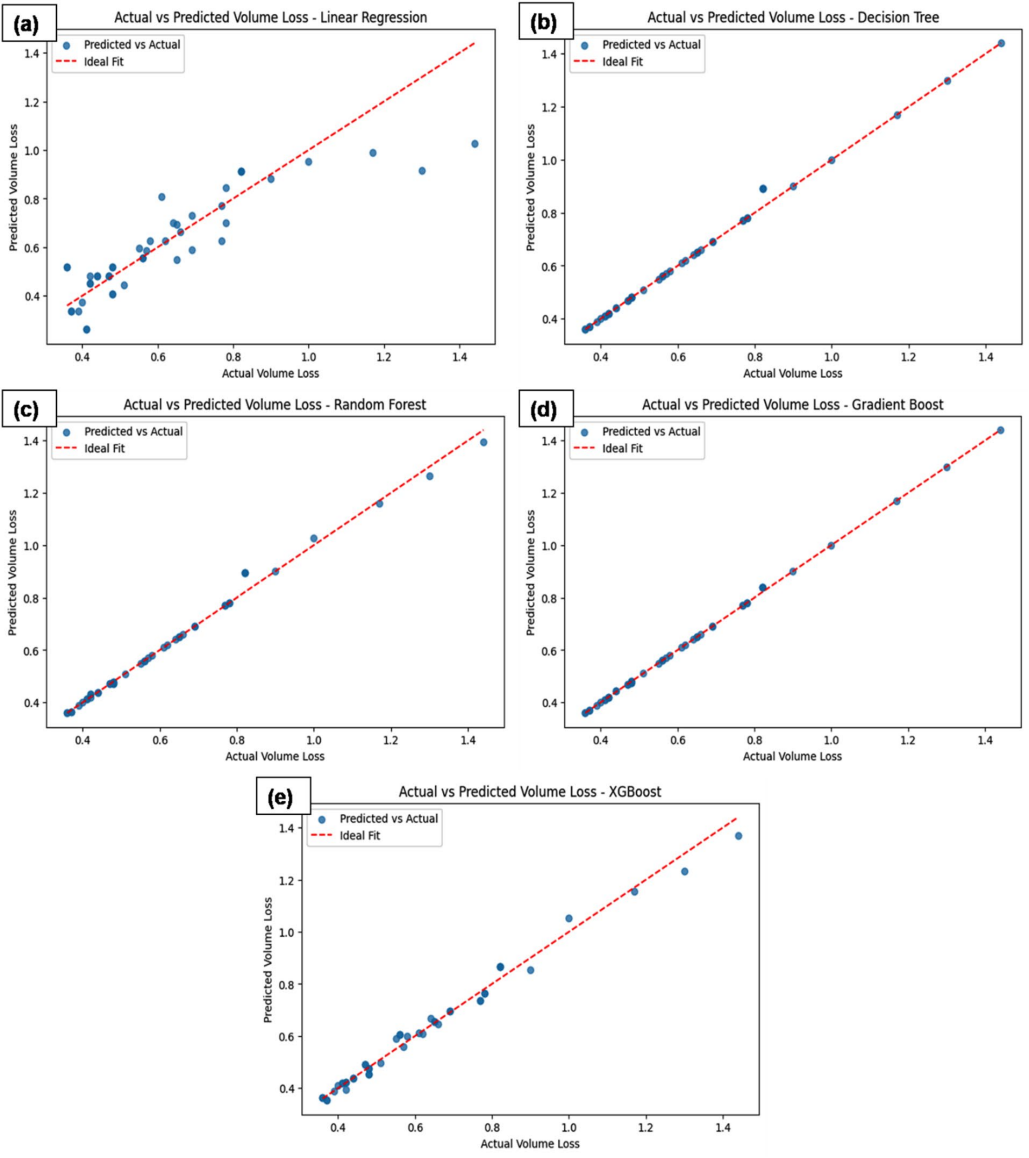
(**a**-**e**) Comparative analysis of the actual and predicted volume loss for different ML models.

Figure 11 presents a comparative analysis of actual vs. predicted volume loss for several machine learning models: (a) LR, (b) DT, (c) RF, (d) GB and (e) XGBoost. After hyperparameter tuning, Gradient Boosting achieved an R² of 0.9987 and RMSE of 0.008755, demonstrating exceptional predictive performance with minimal prediction error. XGBoost closely follows with an R² of 0.9963 and RMSE of 0.014707, highlighting its effectiveness in capturing the underlying patterns and minimizing residuals. The Random Forest model also performed well, yielding an R² of 0.9919 and RMSE of 0.021840, reflecting strong generalization capability, though slightly higher error compared to the ensemble methods. Decision Tree demonstrated satisfactory performance with an R² of 0.9945 and RMSE of 0.018074, though it showed slightly more variability in predictions. In contrast, Linear Regression showed limited improvement after tuning, with an R² of 0.7713 and RMSE of 0.116618, indicating significant limitations in capturing the complexity of the data and producing larger residuals. Overall, Gradient Boosting and XGBoost emerge as the top performers, while Linear Regression lags in predictive accuracy.

**Fig. 12:**
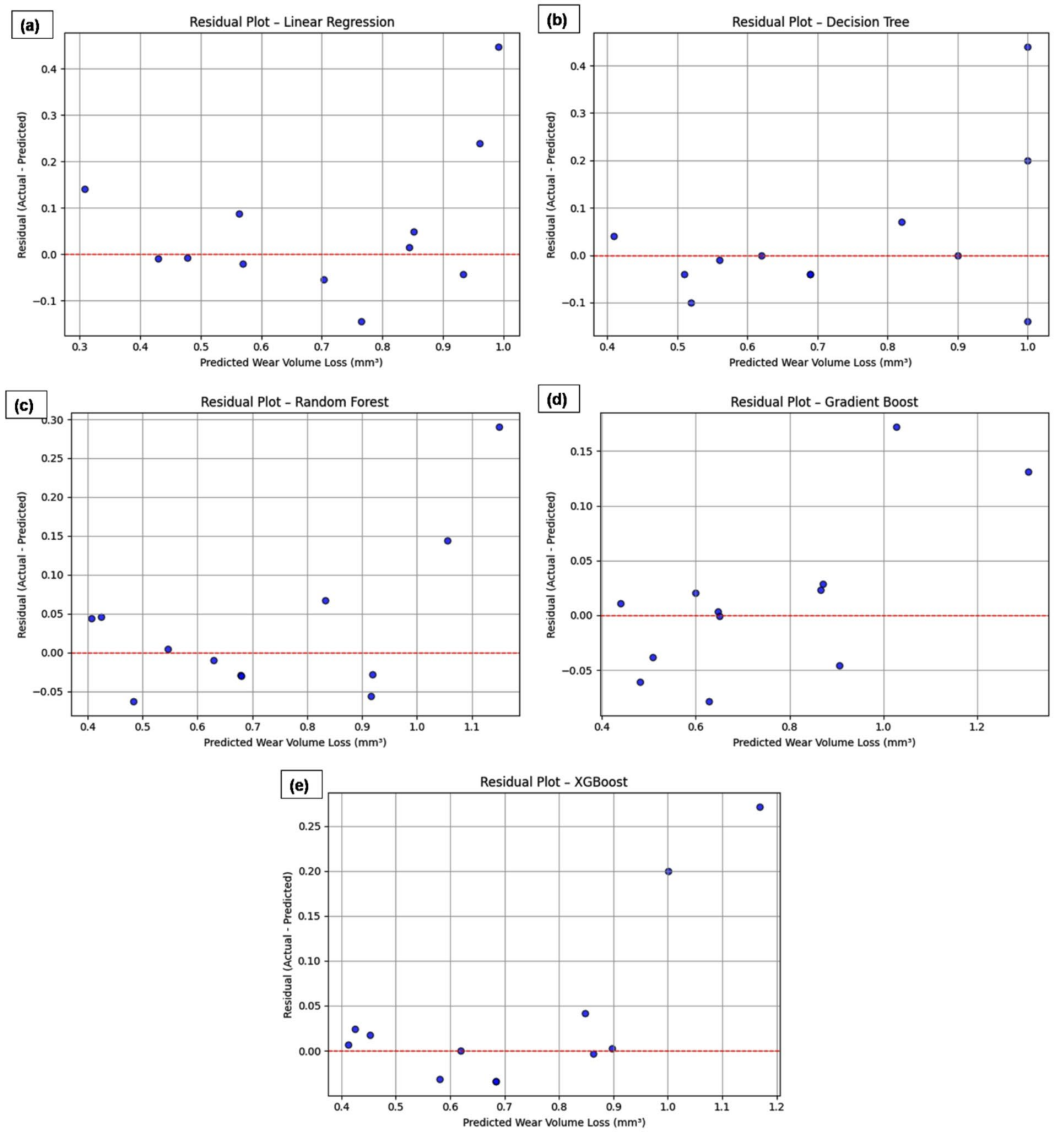
Residual plots for (**a**) Linear Regression, (**b**) Decision Tree, (**c**) Random Forest, (**d**) Gradient Boosting and (**e**) XGBoost models.

To assess the prediction errors in relation to experimental variability, residual plots were generated for each ML model (Fig. 12a–e). Figure 12 presents the residual plots for (a) LR, (b) DT, (c) RF, (d) GB and (e) XGBoost models, which highlight the effectiveness of each model in predicting wear volume loss. The residual plot for Linear Regression shows noticeable spread, indicating poor model fit and higher error, especially for larger predicted values. The DT residuals exhibit a moderate spread with some outliers, suggesting potential overfitting despite an improved fit over Linear Regression. RF, however, shows reduced error with most residuals close to zero, indicating better generalization. GB and XGBoost models provide the most favorable residual plots, with residuals tightly clustered around zero, indicating excellent model fit and minimal error, thus demonstrating their superior ability to capture the underlying data complexity.

**Fig. 13:**
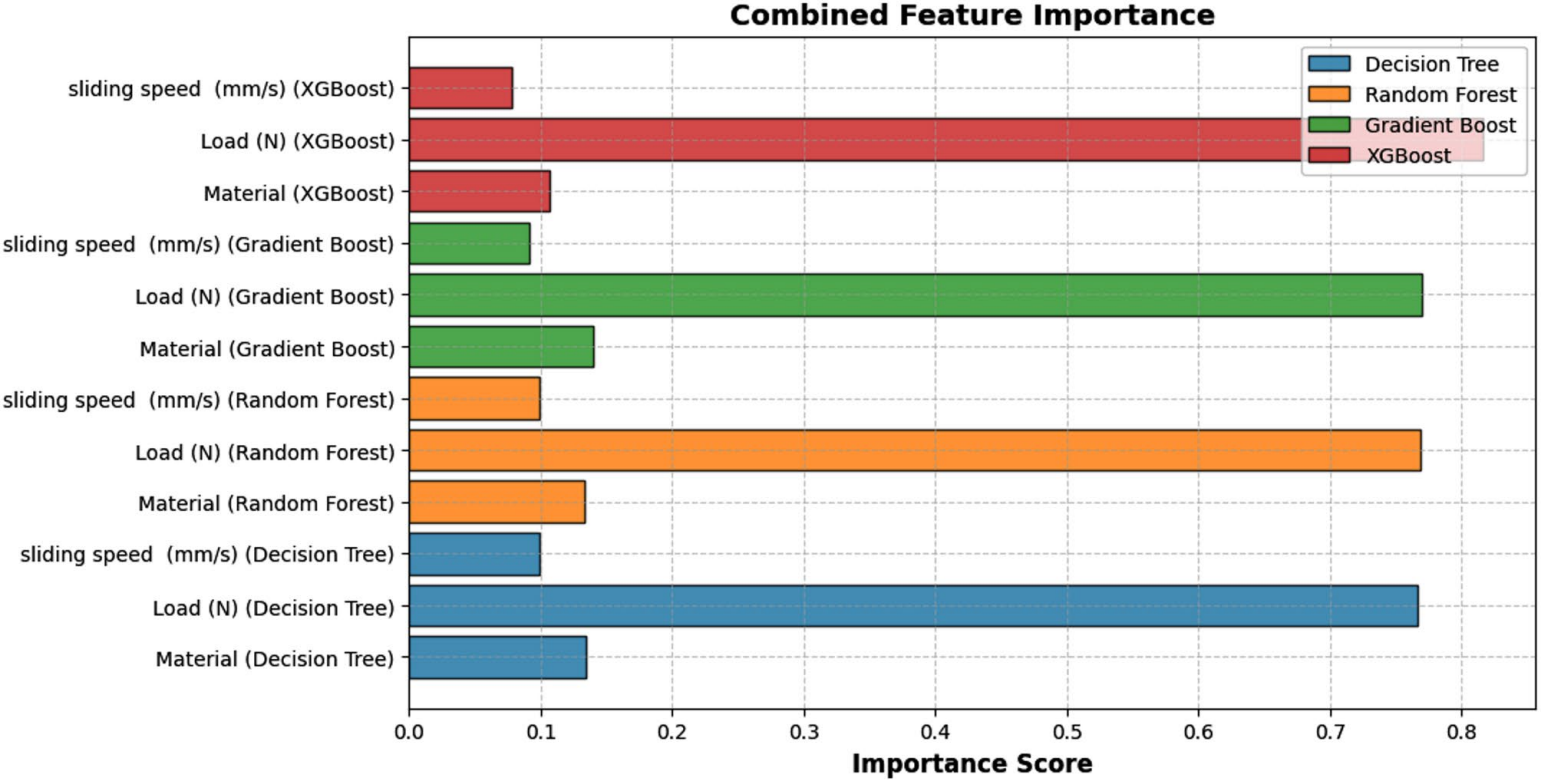
Significance of various features in predicting volume loss.

Figure 13 illustrates the feature importance scores derived from multiple tree-based ML models, providing insight into the relative influence of input variables—load, sliding speed and TiC content—on the predicted wear volume loss. The plot reveals that normal load consistently exhibits the highest importance across all models, emphasizing its dominant role in governing material wear behavior. This aligns with tribological principles, where increased load typically intensifies contact stress and accelerates wear mechanisms such as ploughing, delamination, or adhesion. The reinforcement content (TiC vol%) is the second most influential factor, as it directly affects the composite’s hardness, grain refinement and wear resistance. In contrast, sliding speed shows the least importance, suggesting its secondary role compared to load and material properties in determining volumetric wear.

###  3D Response surface analysis

**Fig. 14:**
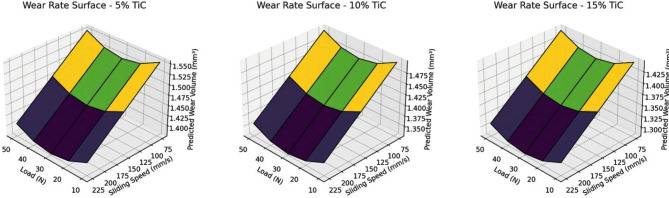
Surface plots for wear rate prediction across load-speed-TiC space.

The 3D surface plots illustrate (Fig. 14) the predicted wear volume behavior of AZ31/TiC composites with 5%, 10% and 15% TiC reinforcement as a function of sliding speed and applied load. Across all compositions, wear volume increases with load due to intensified contact stresses, while it decreases with increasing sliding speed, due to the formation of protective tribolayers at higher velocities. A clear trend of improved wear resistance is observed with increasing TiC content, with the 15% TiC composite showing the lowest predicted wear volume across all conditions. This highlights the effectiveness of TiC in enhancing load-bearing capacity and reducing material removal. The non-linear shape of the surfaces reflects the complex interactions between process parameters, which are accurately captured by the ML model. These plots not only confirm experimental trends but also provide a predictive tool for optimizing wear performance under various operating conditions.

**Table 7:** Comparison with published works.

Source	Material	ML Models	Best Model & Metrics
Singh et al.^51^	Mg–B₄C–Graphite	ANN, RF, KNN, GBM, SVM	GBM, R² ≈ 0.889 (wear), best COF accuracy
Bharath et al.^52^	Mg / eggshell	LR, KNN, RF, Light GBM	Light GBM, highest wear-rate accuracy
Kumar et al.^53^	AZ31/B₄C/GNP	LR, PR, RF, GPR	Polynomial Regression, R² = 0.953, RMSE = 0.103
Current study	AZ31/TiC	LR, DT, RF, GB and XGB	Gradient Boost R² = 0.9987, RMSE = 0.0088 mm³

The present study demonstrates superior wear prediction accuracy compared to existing literature on Mg composites (Table 7). Among all the models evaluated, GB achieved the highest R² value (0.9987) and the lowest RMSE (0.0088), in line with the performance of models such as GB and Light GBM reported in previous works^51– 52^. In contrast, linear regression performed poorly in this study, which differs from its better performance in some earlier studies. This discrepancy emphasizes the significance of dataset-specific hyperparameter tuning. Therefore, the findings of this study further support the reliability and interpretability of Gradient Boost as a robust model for wear prediction in AZ31/TiC composites.

## Conclusions

1. In this study, AZ31 magnesium matrix composites reinforced with 5, 10 and 15 vol% TiC were successfully fabricated using the FSP technique. The influence of TiC content on the microstructure, hardness and dry sliding wear behavior of the composites was systematically investigated.
2. Microstructural analysis confirmed uniform dispersion of TiC particles and significant grain refinement, particularly in the 15 vol% TiC composites, which exhibited an average grain size of ~ 8 μm compared to ~ 60 μm for the unreinforced AZ31 alloy.
3. The Vickers hardness increased progressively with reinforcement content, reaching 116HV for AZ31/15TiC—an 83% improvement over the unreinforced alloy (62 HV). Wear tests revealed that the volume loss decreased significantly with increasing TiC content under all load and speed conditions. For instance, under a 50 N load at 75 mm/s sliding speed, volume loss reduced from 1.45 mm³ (AZ31**)** to 1.0 mm³ (AZ31/15TiC), demonstrating a 31% reduction in wear. At higher speeds and loads, wear resistance further improved due to strain hardening and protective oxide layer formation.
4. ML models were employed to predict wear volume loss based on the experimental dataset comprising 135 data points. Among the five models evaluated, Gradient Boost emerged as the most accurate, achieving an R² score of 0.9987 and RMSE of 0.0087 after hyperparameter tuning. The feature importance analysis showed that normal load was the most influential variable, followed by TiC content and sliding speed.
5. These findings demonstrate the potential of integrating FSP-based composite fabrication with ML for accurate wear performance prediction. The enhanced wear resistance and predictive modeling of AZ31/TiC composites make them promising candidates for lightweight structural components in automotive, aerospace and biomedical applications, where weight reduction and wear resistance are critical. Future work may extend this methodology to hybrid reinforcements and multi-objective optimization using advanced AI-based surrogate models.

## Data Availability

All data generated or analysed during this study are included in this published article.
